# Machine learning for endoleak detection after endovascular aortic repair

**DOI:** 10.1038/s41598-020-74936-7

**Published:** 2020-10-27

**Authors:** Salmonn Talebi, Mohammad H. Madani, Ali Madani, Ashley Chien, Jody Shen, Domenico Mastrodicasa, Dominik Fleischmann, Frandics P. Chan, Mohammad R. K. Mofrad

**Affiliations:** 1 Molecular Cell Biomechanics Laboratory, Departments of Bioengineering and Mechanical Engineering, University of California, 208A Stanley Hall #1762, Berkeley, CA 94720-1762 USA; 2 Department of Radiology, School of Medicine, Stanford University, Stanford, CA USA; 3 Salesforce Research, Palo Alto, CA USA; 4 Molecular Biophysics and Integrative Bioimaging Division, Lawrence Berkeley National Laboratory, Berkeley, CA 94720 USA

**Keywords:** Health care, Medical research

## Abstract

Diagnosis of endoleak following endovascular aortic repair (EVAR) relies on manual review of multi-slice CT angiography (CTA) by physicians which is a tedious and time-consuming process that is susceptible to error. We evaluate the use of a deep neural network for the detection of endoleak on CTA for post-EVAR patients using a novel data efficient training approach. 50 CTAs and 20 CTAs with and without endoleak respectively were identified based on gold standard interpretation by a cardiovascular subspecialty radiologist. The Endoleak Augmentor, a custom designed augmentation method, provided robust training for the machine learning (ML) model. Predicted segmentation maps underwent post-processing to determine the presence of endoleak. The model was tested against 3 blinded general radiologists and 1 blinded subspecialist using a held-out subset (10 positive endoleak CTAs, 10 control CTAs). Model accuracy, precision and recall for endoleak diagnosis were 95%, 90% and 100% relative to reference subspecialist interpretation (AUC = 0.99). Accuracy, precision and recall was 70/70/70% for generalist1, 50/50/90% for generalist2, and 90/83/100% for generalist3. The blinded subspecialist had concordant interpretations for all test cases compared with the reference. In conclusion, our ML-based approach has similar performance for endoleak diagnosis relative to subspecialists and superior performance compared with generalists.

## Introduction

Endovascular aortic repair (EVAR) is the primary treatment for many patients with aortic pathology particularly in the setting of abdominal aortic aneurysm. The procedure has largely replaced the traditional open surgical approach employed in the past which is often associated with increased morbidity and mortality in the peri-operative period^1–5^. Lifelong surveillance imaging is typically performed to evaluate for postoperative EVAR complications which may be asymptomatic and potentially fatal^6^. Endoleak is one of the main and recognized complications associated with EVAR^7^. Endoleak is defined as persistence of blood flow outside the stent graft and within the aneurysm which may lead to growth and subsequent rupture of the aneurysm sac^8, 9^. Computed tomography angiography (CTA) is the standard imaging technique for postoperative surveillance following EVAR^10, 11^. Currently in the routine clinical setting, detection of endoleak requires manual review of multi-slice CTA scans. The interpretation process by humans is tedious, may be subject to error and/or demonstrate variability between human readers.

Machine learning (ML) is an emerging technique which has been increasingly applied to various fields in medicine such as cardiology^12^, radiology^13–15^, ophthalmology^16, 17^, dermatology^18, 19^, and pathology^20, 21^. Machine learning algorithms may learn from examples and respond to new inputs based on their prior training^22^. Machine learning may provide a means to facilitate human CTA endoleak detection in various ways including efficiency, accuracy and standardization of interpretation. The objective of our study is to develop and test a machine learning based model for endoleak detection as well as compare its performance with that of both subspecialist and general diagnostic radiologists.

A substantial amount of labeled segmentation maps is needed to produce a state-of-the-art supervised deep learning segmentation model. Obtaining manual segmentation maps for medical images is a tedious and costly process. Data augmentation is an important and effective method to help capture the complete distribution of possible data. Advanced data augmentation techniques have been shown to provide substantial model performances increases^26^. Studies have also shown the feasibility of getting state-of-the-art performance by augmenting just one image^27^. Data augmentation techniques such as random image rotations and introduction of nonlinear deformations have been shown effective in improving medical segmentation model accuracies^28^. Other augmentation methods involving adding or subtracting regions of images have not been commonly used for natural images because the outcomes appear artificial. However, the addition or subtraction of regions in CT images may be more feasible due to the constraints of CT images compared to natural images. In this study, we present a novel data augmentation method using segmentation maps to augment CT slices by adding and removing regions of the CT slice containing an endoleak.

## Methods

### Data preprocessing

This retrospective study was conducted with the approval of the Stanford Institutional Review Board (IRB) and under a waiver of informed consent. All methods were performed in accordance with relevant guidelines and regulations. Fifty CTA scans from 50 post-EVAR patients with endoleak and 20 CTA scans from 20 post-EVAR patients without endoleak were retrospectively identified. The presence or absence of endoleak in each patient was determined based on the corresponding clinical CTA radiology report dictated by a cardiovascular imaging subspecialty trained diagnostic radiologist. Post-EVAR patients without endoleak are referred to as controls in this study.

The CTA imaging from the positive endoleak cases and negative controls were sent from the picture archiving and communication system (PACS) to TeraRecon (TeraRecon Inc., Foster City, CA) for obtaining de-anonymized DICOM images. The deanonymized CT images included noncontrast, arterial phase, and delayed phase images for each positive endoleak case and control. The de-anonymized images were subsequently transferred to a secure encrypted password-protected server. The DICOM files were then processed into a machine-readable format using Pydicom, flattened, and stored in a storage-efficient manner using HDF5 standards.

CTA scans were split into sets used for training (32 positive endoleak CTAs, 8 control CTAs), validation (8 positive endoleak CTAs, 2 control CTAs), as well as a held-out test subset (10 positive endoleak CTAs, 10 control CTAs). Endoleak regions were labeled for training purposes by manual contouring of individual CT images on all positive endoleak CTAs which was performed by a diagnostic radiologist with cardiovascular imaging subspecialization. Manual segmentation of the endovascular stent lumen and aneurysm sac was also performed for a subset of the controls (10 CTAs). Image pre-processing was performed by thresholding the raw pixel values, mean-centering data, and resizing images.

### Data augmentation

**Figure 1:**
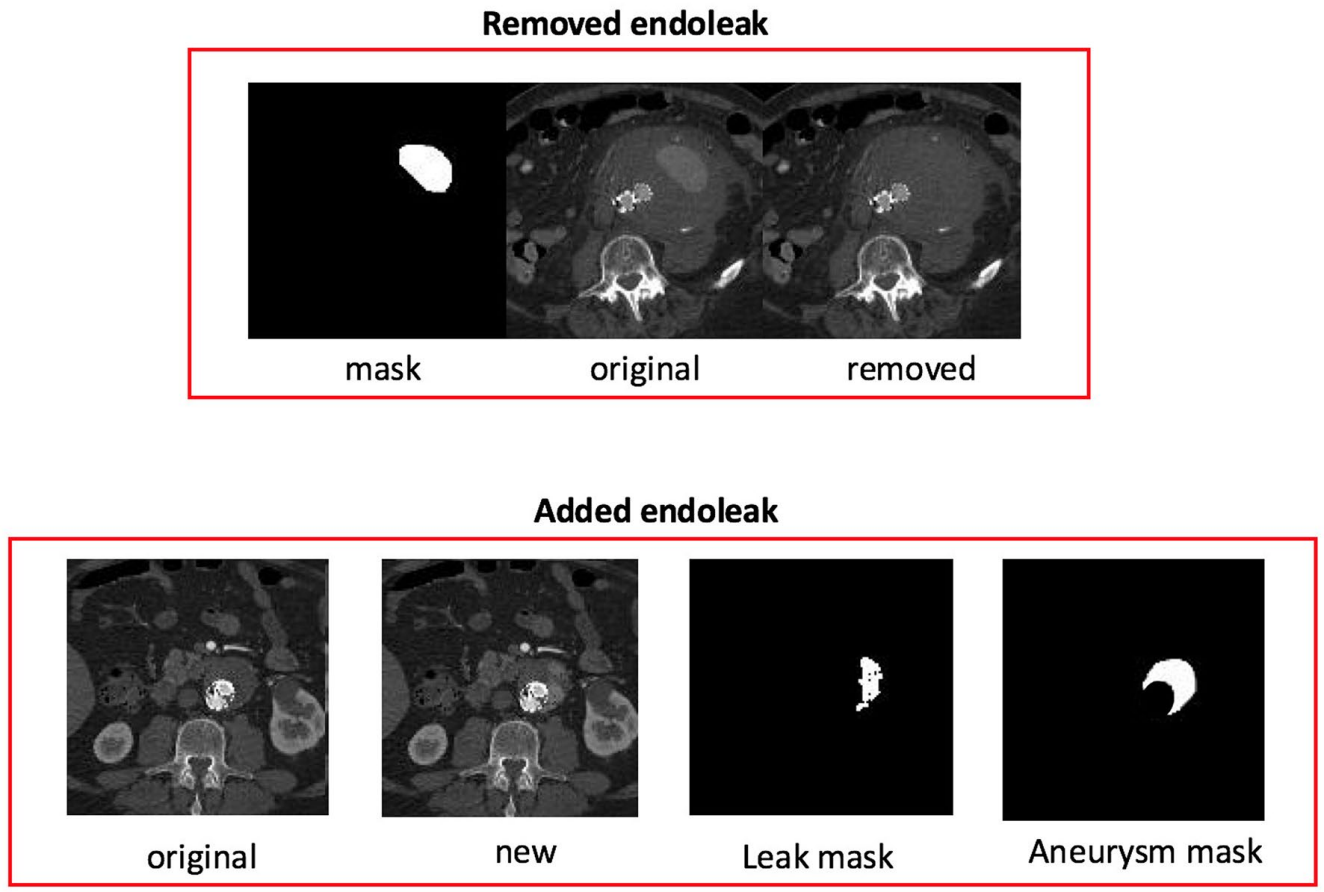
(Top) Overview of the custom data augmentor for removing an endoleak. The CTA slice with a corresponding mask of the endoleak is input into the data augmentor. The data augmentor uses the endoleak mask to remove the endoleak from the CTA slice. (Bottom) Overview of the custom data augmentor for adding an endoleak into an aneurysm sac. The CTA slice with a corresponding mask of the aneurysm sac is input into the data augmentor. The data augmentor uses the aneurysm mask to artificially create a unique and randomly shaped endoleak into the CTA slice.

Standard data augmentation techniques included image rotation and introduction of pixel noise to enhance the training process for the ML model. Additional data augmentation was also performed with a custom developed endoleak augmentation technique referred to as Endoleak Augmentor. Endoleak Augmentor involves the addition and removal of endoleaks on individual CTA images using aneurysm sac and endoleak masks derived from segmentation (Fig. 1) which were then inputted to the ML model for training. Output from the Endoleak Augmentor was verified for adequate addition and removal of endoleaks by a cardiovascular imaging subspecialist radiologist prior to supplying the images to the algorithm for training.

The Endoleak Augmentor was integrated into Keras as a custom data generator class (Algorithm 1). During data generation a batch of labeled data *X* are selected. For each *x*_b_ in the batch we generate transformed version *x’*_b_ depending on the augment ID *l*_b_ (algorithm 1, lines 5, 8 and 12).

Endoleaks are inserted into CT slices using *x’*_b_, *y’*_b_ = augment_adder(*x*_b_, *x*_ab_, *u*_e_, σ_e_) (algorithm 1, line 9). Using the aneurysm sac segmentation map *x*_ab_ as a boundary, an endoleak of random shape is generated and inserted into the aneurysm sac. Pixel values for the endoleak are calculated using a Gaussian distribution of endoleak pixel values calculated from *u*_e_, σ_e_. A new CT slice *x’*_b_ is generated that contains an endoleak.

Endoleaks are removed from CT slices using *x’*_b_, *y’*_b_ = augment_remover(*x*_b_, *x*_eb_, *u*_a_, σ_a_) (algorithm 1, line 13). Using the endoleak segmentation map *x*_eb_ the endoleak pixels are replaced using a Gaussian distribution of aneurysm sac pixel values calculated from *u*_a_, σ_a_. A new CT slice *x’*_b_ is generated with the endoleak removed.

#### Algorithm 1

Endoleak Augmentor takes a batch of labeled CT slices *X* and corresponding augmentation labels l. Depending on the augmentation label, a CT slice will either have an endoleak added, an endoleak removed, or no augmentation. The algorithm produces a collection of updated labeled CT slices *X*’.


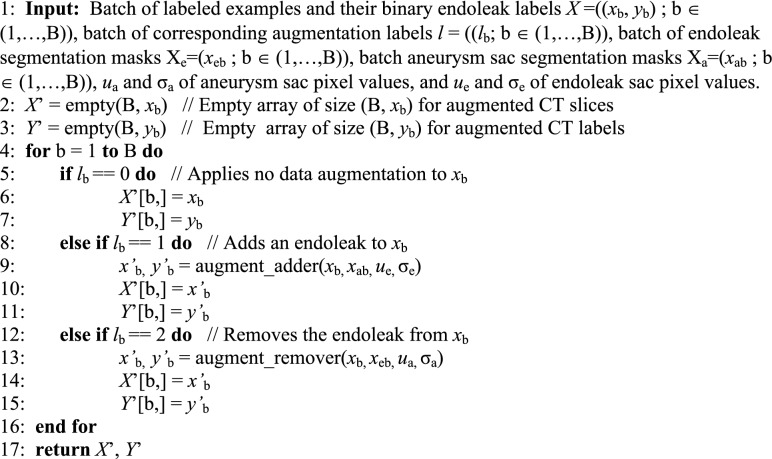


### Model training and evaluation

**Figure 2:**
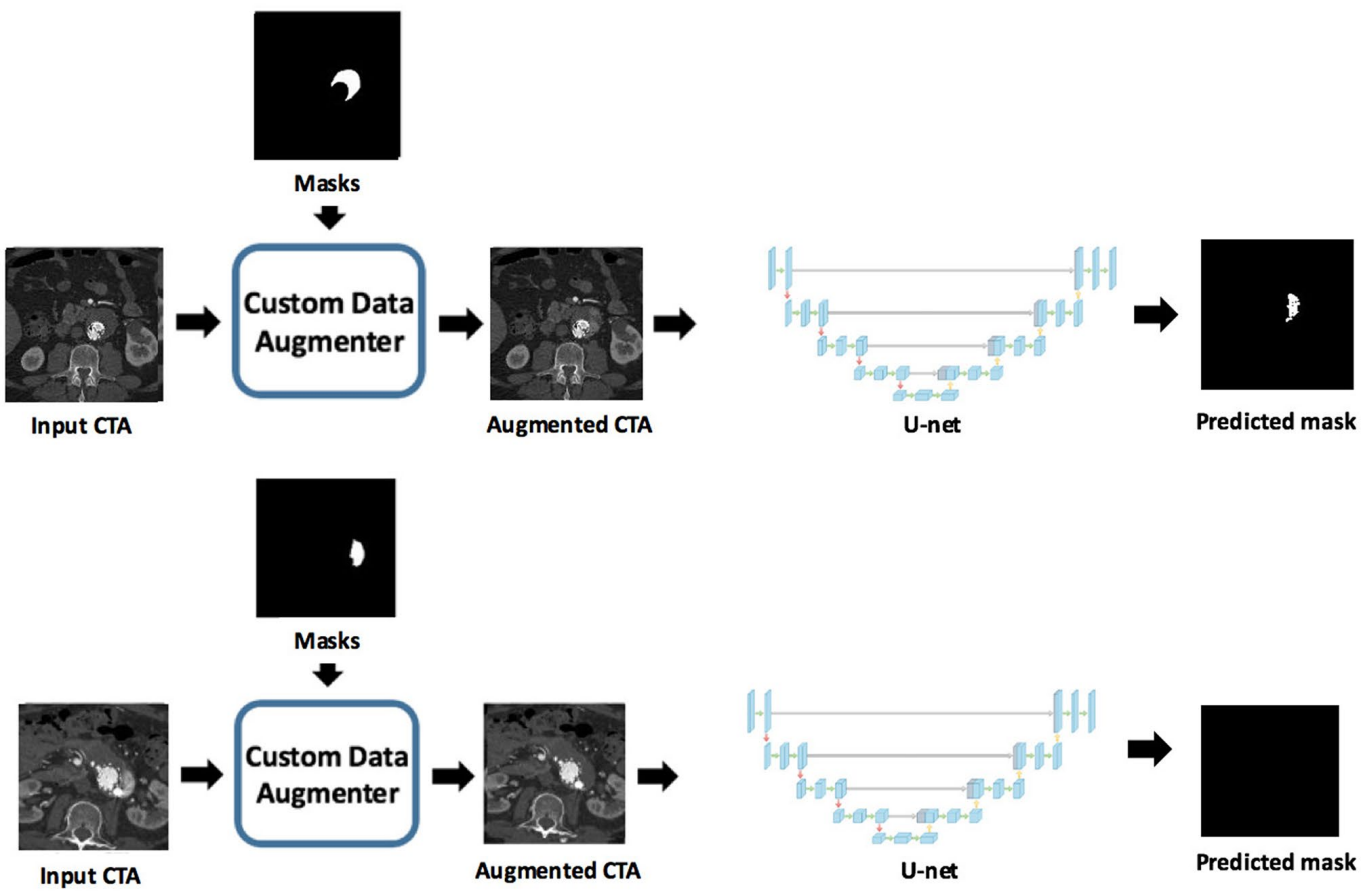
Overview of the pipeline illustrating use of the custom data augmentor and U-net convolutional neural network (CNN).

**Figure 3:**
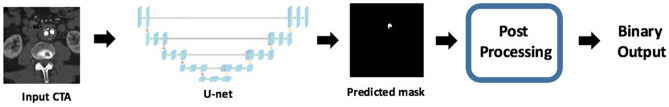
Model evaluation.

Our training set contains a total of 10,539 CTA slices of which 4190 slices contain endoleak segmentation maps and 1532 contain stent lumen and aneurysm sac segmentation maps. A separate validation set containing a total of 1940 CTA slices of which 746 were positive for endoleaks. The training slices are input into the Endoleak Augmentor which transforms the slices and inputs them into our deep learning model. A U-net style model with a convolutional encoder–decoder architecture was used to generate predicted endoleak segmentation maps. Model overview with architecture is depicted in Figs. 2, 3. During training, various hyperparameters were evaluated to improve the model’s prediction performance. Next, the threshold to determine whether a slice is considered yes for endoleak is optimized by picking the probability threshold that maximized the precision, recall and F1 scores of the validation set. Additional post processing logic is implemented to remove false positive predictions that contain very small or very large endoleaks. A final post processing step is performed on all of a patient’s CTA slices to determine if a patient has an endoleak. Testing was performed at a per slice and per case level based on a subset of 10 positive endoleak cases and 10 controls. The same test subset was also reviewed by three additional blinded general diagnostic radiologists and one blinded cardiovascular imaging subspecialty trained radiologist for comparison against the ML model at a per case level.

## Results

Table 1 summarizes the clinical characteristics of the study’s patients. Patients were predominately male and elderly with elevated systolic blood pressures. The most frequent comorbidity among patients was hypertension followed by coronary artery disease with no statistically significant differences between post-EVAR patients with and without endoleak. The majority of patients with endoleak as well as most of the controls underwent EVAR for abdominal aortic or aortoiliac aneurysm (48/50 for the endoleak group, 18/20 for the control group). Isolated iliac artery aneurysm or abdominal aortic dissection was the EVAR indication in 2/50 endoleak group patients and 2/20 control group patients. Prevalence of endoleak types are as follows: type 1 (5/50), type 2 (28/50), type 3 (8/50). Nine of the remaining 50 endoleak group patients had either multiple or indeterminate endoleak types. Embolization material in conjunction with EVAR was present in 10/50 endoleak group patients and 2/20 control group patients. Three of the twenty (15%) of the CTA scans in the test subset had embolization material associated with the endovascular stent graft.

**Table 1:** Patient clinical characteristics.

	Positive endoleak cases (n = 50 patients)	Control cases without endoleak (n = 20 patients)	
	Mean (SD)	Mean (SD)	*P* value
Age (years)	77.4 (4.9)	75.5 (7.1)	0.32
Male/female	5:1	19:1	0.21
Height (cm)	172.8 (9.6)	174.3 (6.5)	0.46
Weight (kg)	80.7 (18.6)	88.3 (15.0)	0.11
BMI (kg/m^2^)	26.9 (4.6)	29.0 (4.3)	0.07
Systolic BP (mm Hg)	138.5 (20.5)	140.0 (21.9)	0.79
Diastolic BP (mm Hg)	73.8 (12.3)	71.7 (14.2)	0.54
Comorbidities	N (%)	N (%)	
HTN	47 (94)	16 (80)	0.08
CAD	23 (46)	13 (65)	0.15
DM	12 (24)	6 (30)	0.60
Stroke/TIA	5 (10)	0 (0)	0.31
CKD	14 (28)	4 (20)	0.49

**Figure 4:**
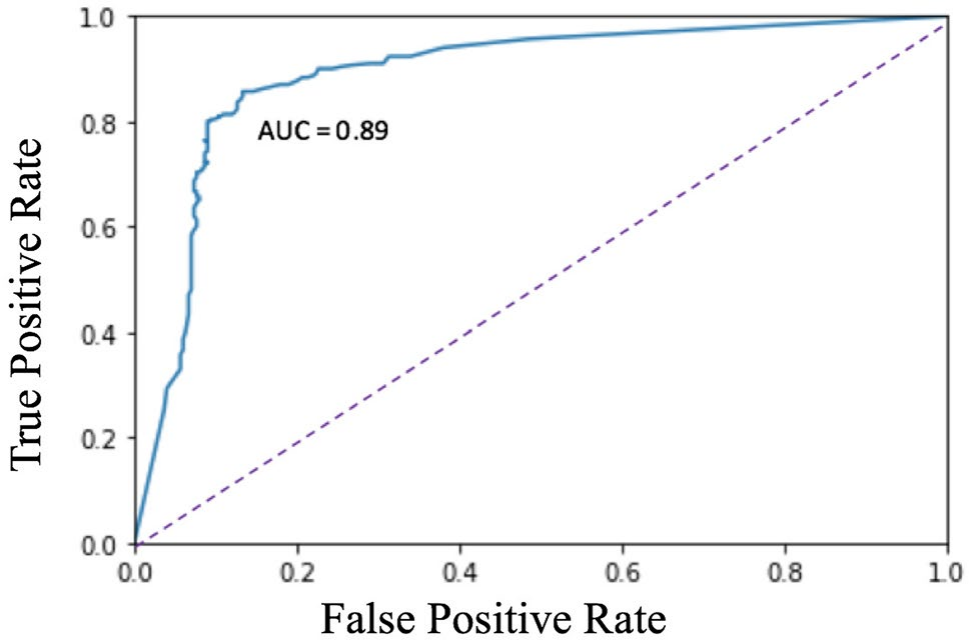
Receiver operating characteristic curve for CT Slice endoleak detection by the machine learning model.

**Figure 5:**
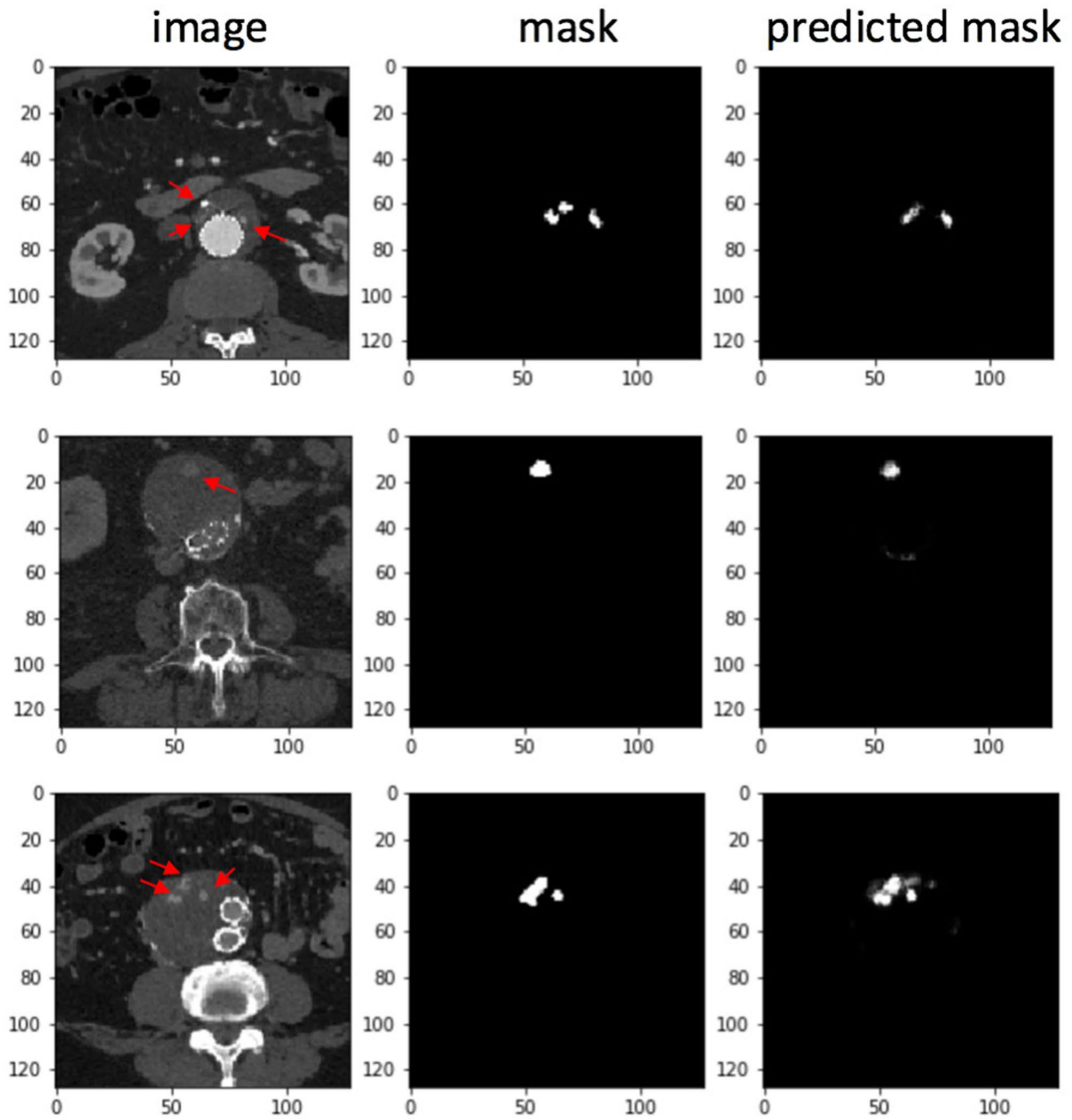
Raw CT slice (left) followed by human annotated segmentation map (middle) and model predicted endoleak map (right).

The area under the curve (AUC) for individual CT slice prediction at a per slice level was 0.89 for a set of 600 CT slices randomly selected from the test subset (Fig. 4). A patient level prediction is then made using an ensemble of all of a patient’s individual CT slice predictions. Performances of the machine learning model and 3 blinded general diagnostic radiologists on a per patient or case level relative to the gold standard interpretation by a cardiovascular imaging subspecialty trained radiologist are shown in Table 2. Accuracy, precision and recall of the ML model was 95%, 90%, and 100% with an AUC of 0.99. The accuracy confidence intervals— as determined by the standard deviation of 1000 bootstrapped sets sampled with replacement—was 11%. Accuracy, precision and recall was 70%, 70% and 70% for the blinded general radiologist 1. Blinded general radiologist 2 had an accuracy of 50%, precision of 50% and recall of 90%. Accuracy, precision and recall for the blinded general radiologist 3 was 90%, 83% and 100%. The blinded subspecialist had interpretations for presence or absence of endoleak that were concordant with the reference clinical radiology report dictated by a cardiovascular imaging subspecialist for all test cases. Examples of masks predicted by the ML model are shown in Fig. 5.

**Table 2:** Performance metrics for the machine learning (ML) model and general radiologists.

	Accuracy (%)	Precision (%)	Recall (%)
ML model	95	90	100
General Radiologist1	70	70	70
General Radiologist2	50	50	90
General Radiologist3	90	83	100

## Discussion

The detection of endoleak following EVAR requires meticulous review of multi-slice CTA scans by humans which can be time-consuming and potentially inaccurate. There has been sparse prior investigation into the application of machine learning in the setting of post-EVAR CTA imaging particularly evaluation for endoleak. One study has demonstrated use of a computer vision algorithm for the segmentation of the inner and outer boundaries of abdominal aortic aneurysms^23^. A recent study by Hahn et al. evaluated the use of a deep learning method for endoleak identification^24^. Our machine learning model accuracy exceeded their study (95% vs. 89%) with an AUC of 0.99 versus 0.94. Hahn et al. reported using the radiology report as the gold standard for endoleak detection with only a subset of 100 CTA images independently read by two human readers consisting of one interventional radiologist and one vascular surgeon. However, the performance metrics of their ML model relative to each of the individual human reader performances for endoleak diagnosis were not stated. In our study in addition to using the radiology report interpreted by cardiovascular imaging subspecialized diagnostic radiologists as the gold standard, we report our machine learning endoleak detection performance along with the individual performances of 3 additional blinded general diagnostic radiologists and 1 additional blinded cardiovascular imaging subspecialty trained radiologist. The purpose of our study design is to provide for further direct comparison at both a machine versus human level and at a human versus human level. Our study raises the possibility that there may be variability even among human readers for endoleak diagnosis, a topic which has not been extensively investigated in the literature^25^. The prior study also excluded CT images with prior embolization although fifteen percent of our test subset CTAs had embolization material which can be encountered in the clinical setting.

Data augmentation is critical to the success of machine learning model performance across domains from computer vision to natural language processing. In this work, we introduce a novel data augmentation technique that can be broadly applied for recognition tasks in medical imaging. This augmentation method provides multiple benefits for training a deep learning model. One benefit is the training set now contains anatomically identical CT slices, one with an endoleak and one without an endoleak. This helps the deep learning model focus on the variation representing an endoleak and not other anatomical differences. Another benefit is the amount of new endoleak CT slices we can generate. For each CTA slice containing an aneurysm sac segmentation map we are able insert many uniquely shaped endoleaks. This increases both the quantity and variance of the training data—ideally improving overall performance and reducing overfitting effects.

Our study has several limitations. Since our data came from a single medical center, the generalizability of the machine learning model may have been limited. Furthermore, our sample sizes including the test subset were small and further studies with more patients are needed for validation of the deep neural network. Lastly, our model uses prediction masks to detect only the existence of an endoleak. However, the endoleak prediction masks can serve as interpretable predictions of the endoleak type and aneurysm size. In future work the quantification of endoleak type and aneurysm size from the prediction masks will be evaluated.

The introduction of machine learning based systems into the clinical setting for post-EVAR surveillance may potentially lead to increased efficiency, accuracy, and greater consistency among readers for post procedural complication detection which could in turn result in improved management of these patients. In summary, this study demonstrates that our machine learning method performance is comparable to that of cardiovascular imaging subspecialist radiologists and superior to that of general radiologists. This raises the possibility that machine learning may eventually assist humans in the interpretation of post-EVAR scans in routine clinical practice.
